# Light Modulates Ethylene Synthesis, Signaling, and Downstream Transcriptional Networks to Control Plant Development

**DOI:** 10.3389/fpls.2019.01094

**Published:** 2019-09-12

**Authors:** Alexandria F. Harkey, Gyeong Mee Yoon, Dong Hye Seo, Alison DeLong, Gloria K. Muday

**Affiliations:** ^1^Department of Biology and Center for Molecular Signaling, Wake Forest University, Winston-Salem, NC, United States; ^2^Department of Botany and Plant Pathology, Purdue University, West Lafayette, IN, United States; ^3^Department of Molecular Biology, Cell Biology and Biochemistry, Brown University, Providence, RI, United States

**Keywords:** ethylene, light, transcriptomic meta-analysis, ethylene response, ethylene biosynthesis, hypocotyl, root

## Abstract

The inhibition of hypocotyl elongation by ethylene in dark-grown seedlings was the basis of elegant screens that identified ethylene-insensitive Arabidopsis mutants, which remained tall even when treated with high concentrations of ethylene. This simple approach proved invaluable for identification and molecular characterization of major players in the ethylene signaling and response pathway, including receptors and downstream signaling proteins, as well as transcription factors that mediate the extensive transcriptional remodeling observed in response to elevated ethylene. However, the dark-adapted early developmental stage used in these experiments represents only a small segment of a plant’s life cycle. After a seedling’s emergence from the soil, light signaling pathways elicit a switch in developmental programming and the hormonal circuitry that controls it. Accordingly, ethylene levels and responses diverge under these different environmental conditions. In this review, we compare and contrast ethylene synthesis, perception, and response in light and dark contexts, including the molecular mechanisms linking light responses to ethylene biology. One powerful method to identify similarities and differences in these important regulatory processes is through comparison of transcriptomic datasets resulting from manipulation of ethylene levels or signaling under varying light conditions. We performed a meta-analysis of multiple transcriptomic datasets to uncover transcriptional responses to ethylene that are both light-dependent and light-independent. We identified a core set of 139 transcripts with robust and consistent responses to elevated ethylene across three root-specific datasets. This “gold standard” group of ethylene-regulated transcripts includes mRNAs encoding numerous proteins that function in ethylene signaling and synthesis, but also reveals a number of previously uncharacterized gene products that may contribute to ethylene response phenotypes. Understanding these light-dependent differences in ethylene signaling and synthesis will provide greater insight into the roles of ethylene in growth and development across the entire plant life cycle.

## Introduction

Plant responses to the gaseous hormone ethylene are an excellent model for studying the relationships between hormone synthesis, signaling, transcriptional changes, and development. The identification of ethylene-insensitive mutants in Arabidopsis using molecular genetics opened a new era in dissecting plant hormone signaling (Bleecker et al., 1988; Guzman and Ecker, 1990). Ethylene-insensitive mutants were identified as lacking the ethylene “triple response” in dark-grown seedlings (short, thick hypocotyl and exaggerated apical hook), remaining tall in the presence of excess ethylene (Alonso et al., 2003; Guo and Ecker, 2003; Yanagisawa et al., 2003). This approach enabled the isolation of mutations affecting the activities of core ethylene response machinery, including receptors, signaling proteins, and transcription factors. The functions of these signaling components, as well as the pathways for ethylene synthesis, have subsequently been assayed in additional tissues beyond dark-grown hypocotyls, demonstrating that many of these proteins function in all tissues and growth conditions, but also revealing branches of the ethylene signaling and synthesis pathways that have distinct roles in light-grown plants and in other developmental stages. In particular, ethylene-responsive transcriptional networks and regulatory controls of ethylene biosynthesis show profound differences between light- and dark-grown tissues. Although some of these differences have been reviewed previously (Rodrigues et al., 2014; Booker and DeLong, 2015; Yoon, 2015; Yu and Huang, 2017), recent studies have identified new mechanisms and yielded insight into light-dependent differences. This review highlights the similarities and differences in light-dependent regulation of ethylene synthesis and response in seedlings grown at a range of light levels, focusing on recent publications establishing that the genetic redundancy in ethylene biosynthetic machinery, ethylene receptors, and transcriptional machinery may allow a complex suite of light-dependent developmental responses to this important hormone.

### Basics of the Ethylene Signaling Pathway

The triple response of dark-grown seedlings was exploited in elegant genetic screens that identified mutants exhibiting either ethylene-insensitivity (*ein* or *etr* mutants) (Bleecker et al., 1988; Guzman and Ecker, 1990; Chang et al., 1993), enhanced ethylene signaling in the constitutive triple response (*ctr*) (Kieber et al., 1993; Huang et al., 2003), or synthesis in the ethylene overproducer (*eto*) mutants (Guzman and Ecker, 1990). The genes responsible for these phenotypes have been cloned and mapped to the ethylene signaling and biosynthetic pathways. The signaling pathway begins with ethylene binding to ER-localized receptor proteins (Kendrick and Chang, 2008), which act as negative regulators of the pathway (Hua and Meyerowitz, 1998). In Arabidopsis, these receptors are ETR1, ETR2, EIN4, ERS1, and ERS2 (Chang et al., 1993; Schaller and Bleecker, 1995; Hua and Meyerowitz, 1998; Sakai et al., 1998), which fall into two subfamilies based on sequence similarity of the ethylene binding domains and the presence of conserved histidine kinase domains (Kendrick and Chang, 2008; Stepanova and Alonso, 2009; Shakeel et al., 2013). When ethylene binds, the receptors are turned off, resulting in decreased activity of the inhibitory CTR1 protein kinase and increased EIN2 output (Kieber et al., 1993; Alonso et al., 1999; Huang et al., 2003; Qiao et al., 2009). C-terminal proteolytic cleavage of EIN2 promotes the nuclear localization of the EIN2 C-terminal proteolytic fragment (EIN2-CEND) (Ju et al., 2012; Qiao et al., 2012; Wen et al., 2012). EIN2-CEND-mediated targeting of *EBF1/2* mRNA to the processing body further enhances signaling output (Li et al., 2015; Merchante et al., 2015). Nuclear EIN2-CEND alters transcription *via* activation of the EIN3 and EIN3-LIKE (EIL1 and EIL2) transcription factors (TFs), which then turn on expression of genes encoding other TFs, such as *ERF1* and *EDF1-EDF4* (Chao et al., 1997; Solano et al., 1998; Alonso et al., 2003; Chang et al., 2013). These core TFs likely work with other TFs as part of a gene regulatory network leading to a diversity of transcriptional responses, which have been characterized in multiple genome-wide transcriptional studies (Stepanova et al., 2007; Chang et al., 2013; Feng et al., 2017; Harkey et al., 2018). Ethylene signaling is also modulated by EIN2-mediated translational regulation (Merchante et al., 2015), as well as F-box dependent proteolysis of EIN2 and EIN3 *via* ETP1/2 and EBF1/2, respectively (Guo and Ecker, 2003; Potuschak et al., 2003; Qiao et al., 2009). EBF1/2 are also destabilized by ethylene in an EIN2-dependent manner, allowing increased accumulation of EIN3 (An et al., 2010).

Ethylene signaling proteins have roles that extend beyond their functions in dark-grown Arabidopsis hypocotyls. Genes encoding these proteins have been found across the plant kingdom (Wang et al., 2015), and the proteins have been shown to function in a diversity of tissues and under a range of light conditions (Lanahan et al., 1994; Binder et al., 2006; Plett et al., 2009; Wilson et al., 2014a). Both CTR1 and EIN2 are required for normal ethylene responsiveness in all light conditions in Arabidopsis, indicating that each of these gene products plays a central and non-redundant role in ethylene signaling, regardless of light conditions. Mutants lacking *CTR1* show constitutive ethylene responses in roots and shoots grown in light or dark (Kieber et al., 1993). Mutations in *EIN2* confer insensitivity to added ethylene in dark-grown hypocotyls (Alonso et al., 1999), light-grown rosettes (Kieber et al., 1993), light-grown hypocotyls (Smalle et al., 1997), and roots of dark-grown (Stepanova et al., 2005) and light-grown seedlings (Negi et al., 2008; Harkey et al., 2018).

Ethylene receptors are members of a conserved multi-gene family (Shakeel et al., 2013). As these receptors function as negative regulators, dominant gain-of-function (GOF) mutations, such as *etr1-1* and *etr1-3* in Arabidopsis (Bleecker et al., 1988; Guzman and Ecker, 1990; Chang et al., 1993) and *Neverripe* in tomato (Wilkinson et al., 1995), yield ethylene-insensitive plants. In contrast, null or loss-of-function (LOF) alleles can confer constitutive ethylene response phenotypes (Hua and Meyerowitz, 1998; Shakeel et al., 2013). In Arabidopsis, the five ethylene receptors have been shown to have distinct roles that are tied to specific developmental responses (Shakeel et al., 2013), some of which can be studied only in older plants, which are necessarily grown in light. Similarly, the tomato *Neverripe* gene belongs to a seven-member ethylene receptor gene family and the *Neverripe* mutant carries a GOF mutation that confers ethylene insensitivity in phenotypes observed in both light and dark conditions (e.g., fruit ripening, hypocotyl triple response, and root development) (Wilkinson et al., 1995; Negi et al., 2010; Klee and Giovannoni, 2011). Tomato plants with knockdown of mRNA encoding receptors have also revealed distinct functions for two tomato ethylene receptors (Kevany et al., 2007). In the sections below, we highlight studies that have revealed differences in ethylene responses that are influenced by light and developmental stage, and which require distinct ethylene signaling or synthesis machinery.

### Basics of the Ethylene Biosynthesis Pathway

The enzymatic steps of the ethylene biosynthetic pathway were uncovered in fruit; subsequent work in fruit and in dark-grown Arabidopsis seedlings identified a conserved biosynthetic pathway and revealed important regulatory mechanisms that control pathway activity (Adams and Yang, 1979; Yang and Hoffman, 1984; Booker and DeLong, 2015; Yoon, 2015). The simple and highly conserved pathway has only two committed steps: conversion of S-adenosyl-l-methionine (SAM) to 1-aminocyclopropane-1-carboxylic acid (ACC) by ACC synthase (ACS), followed by conversion of ACC to ethylene by ACC oxidase (ACO) (Houben and Van de Poel, 2019). ACS has been a primary target for researchers interested in understanding regulation of ethylene biosynthesis, as this enzyme catalyzes the first biosynthetic step, which is frequently described as the rate-limiting step (Adams and Yang, 1979; Yang and Hoffman, 1984). *ACS* gene families in land plants encode isozymes belonging to three classes, type-1, type-2, and type-3 (El-Sharkawy et al., 2008; Lin et al., 2009; Booker and DeLong, 2015; Zhu et al., 2015; Lee et al., 2019). The evolution and regulation of ACO, including consideration of conditions under which ACO activity is limiting for ethylene production, have been recently reviewed (Houben and Van de Poel, 2019). There are both transcriptional and post-translational mechanisms that control which ACS and ACO isozymes are expressed and active, leading to distinct enzyme populations in tissue- and developmental stage-specific contexts (Booker and DeLong, 2015; Houben and Van de Poel, 2019). Positive feedback loops, largely driven by transcriptional controls of these biosynthetic enzymes, drive dramatic increases in ethylene production to accelerate fruit ripening (Klee and Giovannoni, 2011). This review will examine new insight into the molecular mechanisms by which ethylene synthesis is modulated by light levels at both transcriptional and post-translational levels.

## Light-Dependent and -Independent Ethylene Responses

### Ethylene Effects in Hypocotyls Are Opposite in Light and Dark

**Figure 1:**
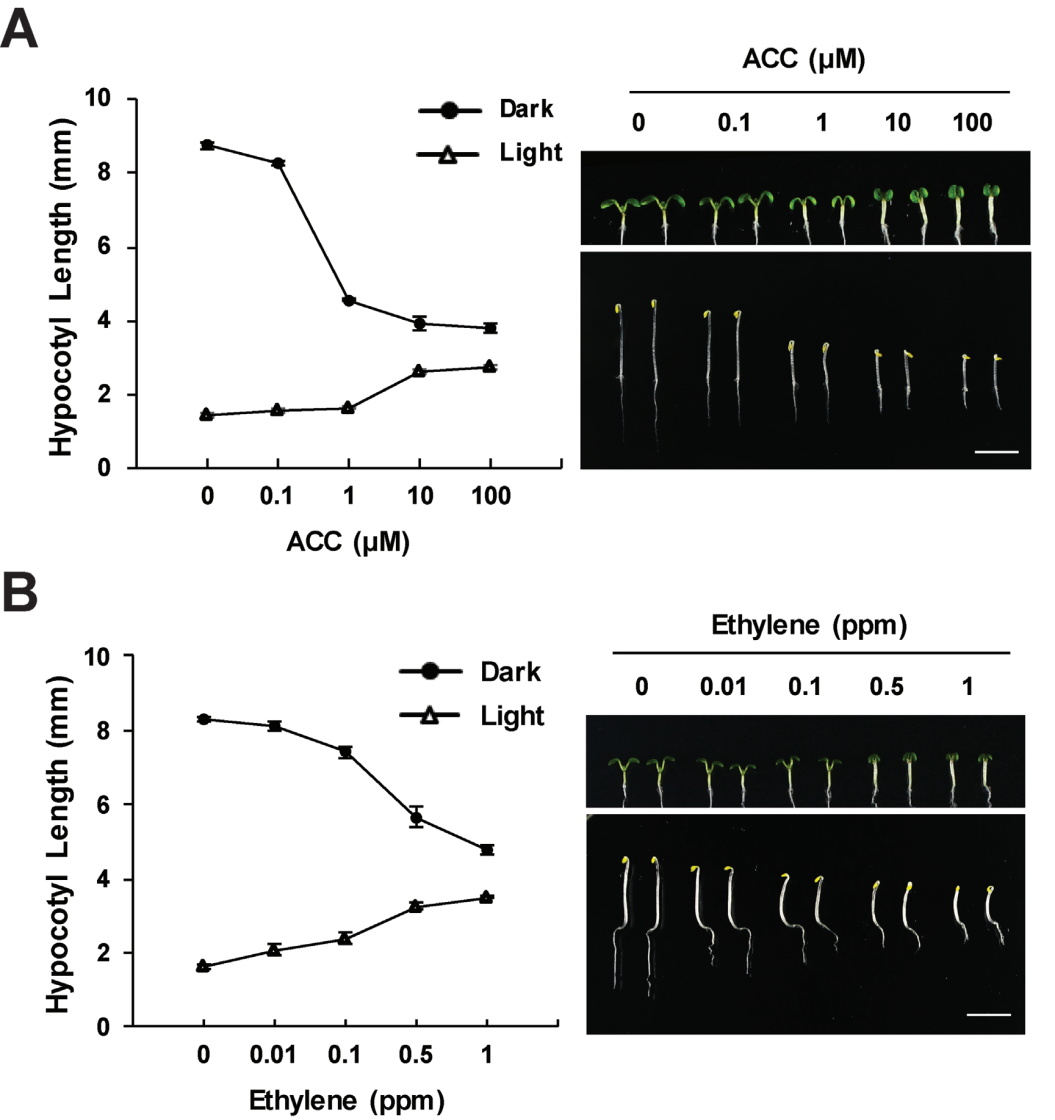
Ethylene and ACC inhibit hypocotyl elongation in the dark and increase elongation in the light. Wild-type seedlings were grown on media containing the indicated concentrations of ACC or on control media and treated with ethylene gas for 3 days in the dark or 5 days in light. The effects of **(A)** ACC or **(B)** ethylene on hypocotyl growth in dark and light conditions. Images generated by the Yoon lab (Seo and Yoon, 2019), recapitulating previous findings (Bleecker et al., 1988; Guzman and Ecker, 1990; Smalle et al., 1997; Le et al., 2005; Liang et al., 2012). Values shown are the average and SD of three replicates, each containing at least 20 seedlings.

The ethylene response in the hypocotyls of young seedlings is highly dependent on light level. The triple response of etiolated seedlings, including inhibited hypocotyl elongation, is the basis of much of the current molecular insight into ethylene signaling (Bleecker et al., 1988; Guzman and Ecker, 1990). Ethylene treatment under shade covering, rather than complete darkness, also leads to decreased hypocotyl growth (Das et al., 2016). The hypocotyl response to ethylene is coordinated with light-dependent hypocotyl elongation changes during photomorphogenesis (Yu and Huang, 2017). Light inhibits hypocotyl elongation, which is important as plants growing in soil transition to light (Montgomery, 2016). In opposition to the effect of ethylene in the dark, light-grown Arabidopsis seedlings show increased hypocotyl elongation in response to ethylene (Smalle et al., 1997; Le et al., 2005; Das et al., 2016; Seo and Yoon, 2019), as illustrated in **Figure 1**. In both light and dark, the ACC or ethylene response is tied to differences in cell expansion (Smalle et al., 1997; Seo and Yoon, 2019). These light-dependent differences have more frequently been reported in response to treatment with the ethylene precursor, ACC (Smalle et al., 1997; Le et al., 2005), but ethylene yields the same light-dependent increases in elongation (**Figure 1**), and ethylene-insensitive mutants are shorter than wild-type in the light (Le et al., 2005). Intriguingly, the nutrient content of the growth media affects the ethylene response in light-grown, but not dark-grown, seedlings (Smalle et al., 1997; Collett et al., 2000).

Another striking feature of the ethylene triple response in etiolated seedlings is the accentuation of the apical hook. As part of photomorphogenesis, the apical hook opens and cotyledons expand, so it is important to ask whether this ethylene response, like hypocotyl elongation, is also light dependent (Bleecker et al., 1988; Raz and Ecker, 1999; Mazzella et al., 2014; Van de Poel et al., 2015). The formation of apical hooks in etiolated seedlings protects the shoot apical meristem during growth through soil, and ethylene build-up in denser soil exaggerates this hook to assist in emergence (Zhong et al., 2014; Shi et al., 2016a). Ethylene insensitive mutants with receptor and signaling defects show impaired hook formation, while the *ctr1-1* null mutant has an exaggerated hook (Abbas et al., 2013). Localized accumulation of ACO across the hook may also contribute to hook maintenance in dark-grown seedlings (Peck et al., 1998; Raz and Ecker, 1999). Mutants with elevated ethylene synthesis show enhanced hook formation (Guzman and Ecker, 1990). A central feature of ethylene-accentuated hook formation is crosstalk with auxin. Asymmetries in auxin synthesis and auxin transport, which lead to accumulation of growth-inhibiting auxin levels on the inside of the hook, are enhanced by ethylene treatment (Vandenbussche et al., 2010; Zádníková et al., 2010). The process of hypocotyl hook opening in response to light is also ethylene regulated (Vandenbussche et al., 2010; Zádníková et al., 2010; Van de Poel et al., 2015). In dark-grown seedlings, the *ein3-1 eil1-1* double mutant has enhanced hook opening, while an EIN3 overexpression line has a tightly closed, exaggerated hook like *ctr1-1*, and shows delayed hook opening in the light (Zhang et al., 2018), consistent with ethylene negatively regulating hook opening in both light and dark.

### Ethylene Modulates Light-Dependent and Light-Independent Root Development

In seedling roots, ethylene and ACC inhibit elongation in both light and dark conditions (Rahman et al., 2001; Ruzicka et al., 2007; Stepanova et al., 2007; Swarup et al., 2007; Negi et al., 2008; Negi et al., 2010; Strader et al., 2010) while enhancing root hair initiation (Cutter, 1978; Tanimoto et al., 1995; Pitts et al., 1998; Dolan, 2001; Rahman et al., 2002; Strader et al., 2010). In both light- and dark-grown seedlings, these root responses to ethylene are lost in ethylene-insensitive *etr1-3*, a dominant gain of function (GOF) receptor mutant, and in the *ein2-5* signaling mutant (Ruzicka et al., 2007; Swarup et al., 2007; Negi et al., 2008; Lewis et al., 2011a). These effects on root elongation are tied to auxin and ethylene cross-talk in a light-independent fashion. Ethylene enhances auxin synthesis, transport, and signaling to control root development (Stepanova et al., 2005; Ruzicka et al., 2007; Stepanova et al., 2007; Swarup et al., 2007; Negi et al., 2008; Stepanova et al., 2008; Lewis et al., 2011a; Muday et al., 2012).

In contrast, the inhibitory effect of ethylene and ACC on lateral root (LR) formation in Arabidopsis and tomato has been examined only in light-grown seedlings, as LRs do not form in roots of dark-grown seedlings (Ivanchenko et al., 2008; Negi et al., 2008; Negi et al., 2010; Lewis et al., 2011b; Lewis et al., 2011a). Ethylene and ACC block early stages of LR initiation (Ivanchenko et al., 2008). As with the inhibition of root elongation, ethylene inhibits LR formation by modulating auxin synthesis, signaling, and transport, which control this process (Stepanova et al., 2007; Muday et al., 2012). Similarly, the effects of ethylene and ACC on root gravitropism and root waving, which have been assayed only in light-grown seedlings, also are blocked in the ethylene signaling mutants *ein2-5* and the GOF *etr1-3* receptor mutant (Buer et al., 2003; Buer et al., 2006). Overall, published data support a light-independent function of the EIN2 protein in ethylene signaling in roots (Ruzicka et al., 2007; Stepanova et al., 2007; Swarup et al., 2007; Negi et al., 2008; Lewis et al., 2011a). However, these data do not reveal which specific receptors function in the roots, because the use of GOF mutants (like *etr1-1* and *etr1-3*) can perturb the functions of the entire receptor family (Chang et al., 1993; Shakeel et al., 2013). Using LOF alleles in each receptor subtype is a powerful strategy to resolve the specific function of the family of ethylene receptors; this approach has been used to understand ethylene-regulated growth and development in a light-dependent context, as discussed below.

## Mechanistic Connections Between Light Response and Ethylene Biosynthesis

Changes in ethylene synthesis in response to changing light levels have been reported in many different species and under many different growth conditions, with dramatically varying results. The ability of light to modulate ethylene synthesis was reported half a century ago, when a single dose of red light was shown to decrease ethylene levels in etiolated pea seedlings in a far-red reversible manner, suggesting that phytochrome negatively controls ethylene biosynthesis (Goeschl et al., 1967). Conversely, high-intensity illumination of green seedlings induced an increase in ethylene synthesis, demonstrating a positive effect of light on ethylene production (Weckx and Van Poucke, 1989). Subsequent studies have confirmed that the effect of light on ethylene synthesis is complex and context-dependent (Foo et al., 2006; Khanna et al., 2007; Jeong et al., 2016; Song et al., 2018), and is also affected by crosstalk with other plant hormone response pathways (Vandenbussche et al., 2003; Arteca and Arteca, 2008; Muday et al., 2012; Lee et al., 2017). For instance, etiolated Arabidopsis seedlings show age- and light-dependent increases in ethylene biosynthesis with higher levels in the light; increased ethylene production is detectable as rapidly as 4 h after transfer to light, but becomes more dramatic with increasing time in light (Seo and Yoon, 2019). As discussed below, these effects are mediated at both the transcriptional and post-translational levels, and although much work has focused on regulation of ACS expression and activity, additional data reveal light-dependent effects on regulation of ACO function.

### Light-Mediated Transcriptional Regulation of ACS and ACO

Regulation of ethylene synthesis *via* alteration of *ACS* and/or *ACO* gene expression is a primary mechanism through which differences in the quality, quantity, or periodicity of light modulate ethylene production and signaling outputs to coordinate plant growth and development (Yamagami et al., 2003; Tsuchisaka and Theologis, 2004; Wang et al., 2005). The combinatorial effects of light with phytohormones and biotic or abiotic stresses add further complexity to light-mediated control of ethylene biosynthesis. For example, IAA treatment induces expression of Arabidopsis *ACS* genes in seedlings grown in darkness or in constant light, but this induction is less dramatic in plants grown with a light/dark cycle (Rashotte et al., 2005). Furthermore, light differentially influences the transcript levels of various *ACS* genes, depending on the developmental stage and the length of light treatment (Seo and Yoon, 2019). The mRNA levels of a subset of type-1 and type-2 *ACSs* (*ACS6* and *ACS5*, *8*, and *9*, respectively) declined rapidly and steeply after etiolated seedlings were transferred to light, and these transcript levels remained low for 5 days. Meanwhile, *ACS2* (type-1) and *ACS4* (type-2) showed gradual increases in their transcript levels after light exposure (Seo and Yoon, 2019). Together, these data suggest distinct roles for ACS isozymes depending on the light conditions, with *ACS5*, *6*, *8*, and *9* playing the primary roles in dark-grown seedlings, while expression of *ACS2* and *ACS4* is implicated in controlling ethylene production in the light.

Analysis of light signaling mutants and transgenic lines expressing light signaling components has also provided insight into the light-mediated regulation of ethylene biosynthesis. Mutations in the phytochrome genes *PHYA* and *PHYB* increased ethylene biosynthesis in pea, consistent with a negative effect of light on ethylene synthesis, with a more profound effect observed in the *phyA* mutant (Foo et al., 2006). Intriguingly, in Arabidopsis and sorghum, *phyA* mutants show less profound increases in ethylene biosynthesis than do *phyB* mutants, indicating species-specific functions of these photoreceptors in controlling ethylene levels. Similarly, transgenic lines overexpressing Arabidopsis PHYTOCHROME-INTERACTING FACTOR5 (PIF5), a basic helix-loop-helix transcription factor that specifically interacts with the photoactivated form of PhyB, showed a marked increase in ethylene production in the dark that is correlated with increased abundance of *ACS4*, *ACS8*, and other *ACS* transcripts (Khanna et al., 2007). Although the *pif1 pif3 pif4 pif5* (*pifq*) mutant initially produced less ethylene than wild-type seedlings, consistent with the higher ethylene levels in *PIF5* overexpression lines, at later time-points the *pifq* mutant showed higher ethylene production (Jeong et al., 2016), indicating a developmental stage-dependent role of PIFs in controlling ethylene biosynthesis.

The regulation of *ACO* gene expression has received much less study than that of *ACS* (Houben and Van de Poel, 2019), yet the levels of *ACO* transcripts are also regulated by light and other factors that control pathway activity (Argueso et al., 2007; Rodrigues et al., 2014). In tomato fruits, *ACO1* is upregulated by pulses of white light (Scott et al., 2018). Classic work demonstrated that *ACO* expression is both a driver of ethylene production and a reporter for ethylene response in etiolated tissues (Peck and Kende, 1995; Kim et al., 1997), creating a positive feedback loop. *ACO* transcript increases have also been reported after ACC treatment of aerial tissues of light-grown seedlings (Zhong and Burns, 2003). The meta-analysis discussed below provides strong support for this feed-forward mechanism. Furthermore, when ACS activity is elevated during climacteric ripening in tomato or banana fruits (and during flooding stress), ACO activity becomes rate limiting, and ACO expression is up-regulated (Ruduś et al., 2013; Xiao et al., 2013; Houben and Van de Poel, 2019). This suggests that one role of the feed-forward mechanism is to “clear” excess ACC when ACO activity limits ethylene production.

### Light-Mediated Post-Translational Control of ACS and ACO Activity

An early study suggested that light regulates ethylene biosynthesis by altering stability/activity of ACS isozymes (Rohwer and Schierle, 1982). More recent work has confirmed that light modulates ethylene biosynthesis *via* post-translational mechanisms including reversible phosphorylation and protein turnover (Steed et al., 2004; Chae and Kieber, 2005; Yoon and Kieber, 2013b; Zdarska et al., 2015; Seo and Yoon, 2019). Post-translational regulation of ACS is largely dependent on the regulatory motifs located in the C-terminus of ACS proteins (Chae and Kieber, 2005). All three ACS types contain a well-conserved N-terminal catalytic domain, whereas the C-termini vary among ACS isoforms. Type-1 ACSs (ACS1, 2, and 6 in Arabidopsis) possess phosphorylation target sites for mitogen-activated protein kinases (MAPKs) and calcium-dependent protein kinases (CDPKs) (Tatsuki and Mori, 2001; Hernández Sebastià et al., 2004; Liu and Zhang, 2004). Type-2 ACSs (ACS4, 5, 8, 9, and 11 in Arabidopsis) contain a phosphorylation site for CDPKs and a unique regulatory motif called Target of ETO1 (TOE) in the C-terminus. The TOE motif is the binding site for ETHYLENE OVERPRODUCER1 (ETO1) and its two paralogs, ETO1-LIKE1 and 2 (EOL1 and EOL2). ETO1/EOL1/EOL2 are BTB/TRP-containing E3 ligases that control the degradation of type-2 ACS proteins *via* the 26S proteasome (Yoshida et al., 2005). In contrast to both type-1 and type-2 ACSs, the single type-3 ACSs does not contain known regulatory motifs in the C-terminus, but as discussed below, an N-terminal motif may control the stability of Arabidopsis ACS7 (Xiong et al., 2014), a sole type 3 isozyme in Arabidopsis.

The protein stability of all three ACS isozyme types is regulated by 14-3-3 proteins (Yoon and Kieber, 2013a). 14-3-3 proteins are an evolutionarily well-conserved family of regulatory proteins involved in numerous cellular processes such as cell cycle regulation, cell division, cell metabolism, proliferation, and protein oligomerization and localization (Dougherty and Morrison, 2004; Darling et al., 2005; Oecking and Jaspert, 2009; Freeman and Morrison, 2011). 14-3-3 activity influences ethylene biosynthesis by destabilizing ETO/EOL proteins and by stabilizing ACS proteins in an ETO/EOL-independent manner (Yoon and Kieber, 2013a). The range of light-dependent developmental phenotypes observed in 14-3-3 LOF mutants (Pnueli et al., 2001; Mayfield et al., 2007; Tseng et al., 2012; Adams et al., 2014) suggests interaction with multiple light signaling components. Although there is no direct evidence that light regulates interactions between 14-3-3 proteins, ACS isozymes, and ETO/EOLs, the 14-3-3s proteins are logical candidates to mediate crosstalk between light signaling and ethylene biosynthesis pathways.

Light-dependent post-translational control of ACS5 (and perhaps other type-2 ACSs) and the associated increase in ethylene production are critical for regulating hypocotyl elongation during the dark-to-light transition. Intriguingly, PIF3 may be involved in this process (Seo and Yoon, 2019). As described above, PIF3 is required for ethylene-induced stimulation of hypocotyl elongation in the light, and ethylene treatment specifically antagonizes light-induced degradation of PIF3 (Zhong et al., 2012). Light-induced stabilization of type-2 ACS enzymes should lead to increased ethylene production, which may play a role in PIF3 stabilization, thereby driving ethylene-induced hypocotyl elongation in the light (Seo and Yoon, 2019). PP2A is another regulatory component that contributes to post-translational regulation of ACS stability. Genetic analysis indicated that PP2A-mediated dephosphorylation negatively controls the protein stability of ACS6 in the dark, but has a much weaker effect on ethylene production in the light (Skottke et al., 2011). Paradoxically, the stability of ACS5, a type-2 isozyme, is positively regulated by PP2A; differential effects on the two isozyme types likely accounts for the lesser effect of PP2A inhibition in light-grown plants (Muday et al., 2006; Skottke et al., 2011).

Compared to type-1 and type-2 ACS isozymes, the sole Arabidopsis type-3 isozyme, ACS7, has unique protein stability characteristics; regulation of ACS7 turnover remains somewhat controversial (Lyzenga et al., 2012; Xiong et al., 2014; Lee et al., 2017). Because of the lack of C-terminal regulatory motifs in type-3 ACS, it was thought that these isozymes might be generally stable compared to other ACS isozymes. However, recent work showed that the stability of type-3 ACS is negatively regulated by ubiquitin-dependent turnover mediated by XBAT32, a RING-type E3 ligase (Lyzenga et al., 2012). Moreover, a putative N-terminal degron of ACS7 is active only in light-grown plants (Xiong et al., 2014) and is poorly conserved (Booker and DeLong, 2015). This light-dependent regulation of ACS7 stability may be similar to the turnover regulation of type-2 ACS, allowing the fine-tuning of ethylene production to impose transient growth control under changing conditions. Considering the regulatory role of the N-terminal domain in ACS7, it may be important to revisit the question of N-terminal motifs that could be involved in regulating the stability of other ACS proteins in response to various stimuli, including light.

The post-translational modifications of ACO have been examined in less detail than those that regulate ACS activity. However, recent work has identified several post-translational mechanisms for controlling ACO activity, including glutathionylation (Dixon et al., 2005) and sulfhydration of cysteine residues on ACO (Friso and van Wijk, 2015). While the effect of glutathionylation on ACO activity has not been reported, S-sulfhydration of LeACO1 and LeACO2 results in a decrease in ACO activity and ethylene production (Jia et al., 2018), establishing an *in vivo* role for post-translational control of ACO. Determining whether these modifications contribute to light-dependent regulation of ethylene production is an open question for future research.

## Mechanistic Connections Between Light Response and the Ethylene Signaling Pathway

### Ethylene Receptor Function Is Dependent on Light and Developmental Context

The five ethylene receptors in Arabidopsis are not functionally equivalent, with sub-functionalization observed for responses in different tissues and developmental stages (as reviewed by Shakeel et al., 2013). This subfunctionalization was revealed though detailed phenotypic analysis of LOF receptor mutants (Wang et al., 2003; Binder et al., 2004; Binder et al., 2006; Qu et al., 2007; Liu et al., 2010; McDaniel and Binder, 2012; Wilson et al., 2014b; Bakshi et al., 2015; Harkey et al., 2018). This sub-functionalization is likely due to diversity in receptor structure and signaling capabilities (O’Malley et al., 2005; Wang et al., 2006; Shakeel et al., 2013; Bakshi et al., 2015). Like the central signaling mutant *ein2-1*, a GOF *etr1-3* mutant was insensitive to ethylene or ACC in seedlings growth in light or dark (Guzman and Ecker, 1990; Roman et al., 1994; Negi et al., 2008). In an examination of nutation of etiolated hypocotyls, ethylene-dependent nutations were lost in the *etr1-7* LOF mutant no other single receptor LOF mutations affected this process (Binder et al., 2004; Binder et al., 2006). In contrast, the function of EIN4 was light-dependent. In dark-grown seedlings the *ein4-1* receptor GOF mutant showed no ethylene response (Roman et al., 1994). When grown in the light, however, *ein4-1* seedlings show a partial response to ACC (Smalle et al., 1997), suggesting differences in this receptor’s role in dark vs. light.

The functional role of the five ethylene receptors has been explored in roots of light-grown Arabidopsis seedlings (Harkey et al., 2018). Transcripts encoding all five ethylene receptors are expressed in roots, and the abundance of transcripts encoding three receptors, *ETR2*, *ERS1*, and *ERS2*, is increased by treatments that elevated ethylene (Hua et al., 1998; Harkey et al., 2018). The GOF *ETR1* mutant (*etr1-3*) is insensitive to the effects of ethylene on root elongation, LR development, and root hair initiation (Negi et al., 2008; Lewis et al., 2011a). Using null mutants in each of the five receptors, the major role of ETR1 in controlling root responses to ACC was reported, with subtle changes in development in null mutants in any of the other receptors (Harkey et al., 2018). Using multiple LOF mutants in two or three receptor genes, minor and redundant roles for *ETR2* and *EIN4* were identified, especially in root hair formation. A triple mutant carrying *etr1-6*, *etr2-3*, and *ein4-4* LOF mutations has short roots, with no LRs and with extreme proliferation of root hairs. All three phenotypes are largely complemented with a genomic copy of ETR1 (Harkey et al., 2018). These results argue that the ETR1 receptor has a predominant role in controlling ethylene-inhibited LR formation, and ethylene-stimulated root hair initiation in light-grown roots, similar to the major role of this receptor in controlling nutations and responses to silver ions (Shakeel et al., 2013). Two specific receptors regulate the size of the root apical meristem, however (Street et al., 2015). In contrast with findings in LRs and root hairs, LOF *etr1-9* or *ers1-3* single mutants showed wild-type meristem size, but the LOF *etr1-9 ers1-3* double mutant exhibited a substantially reduced root apical meristem size, similar to that found in the *ctr1-2* mutant, consistent with multiple receptors controlling this aspect of root development (Street et al., 2015).

The role of specific ethylene receptors in root elongation in dark-grown seedlings has also been reported. Images of dominant GOF mutants in *ETR1*, *ERS1*, *ERS2*, and *EIN4* show roots that appear to be ethylene-insensitive (Hua et al., 1995; Hua et al., 1998). Responses to added ACC were quantified for several *etr1* and *ers1* mutant alleles, which showed reduced sensitivity (Hua et al., 1995). In comparison, the GOF *etr2-1* mutant appears to have an intermediate phenotype, with roots shorter in ethylene than in air, but not as short as wild-type roots in ethylene (Sakai et al., 1998). One study observed that subfamily 2 receptors (*ETR2*, *ERS2*, and *EIN4*) are not required for ethylene root response, as the *etr1-9 ers1-3* double mutant which carries strong LOF alleles has constitutive ethylene signaling, suggesting that the remaining receptors were not sufficient to repress ethylene signaling (Hall et al., 2012). Additionally, complementation with a wild-type copy of *ETR1* was adequate to restore ethylene sensitivity (Hall et al., 2012). Another group assayed phenotypes of receptor mutants in both light and dark (Adams and Turner, 2010), but in the absence of sucrose, which is also known to influence ethylene response (Zhou et al., 1998; Gibson et al., 2001; Yanagisawa et al., 2003; Haydon et al., 2017). Root length in the GOF *etr1-1* mutant was unchanged in response to ethylene under conditions of continuous darkness, but not continuous light. Some differences in the responses of other receptor LOF mutants were observed in dark- versus light-grown seedlings, but all receptors were at least partially required under both conditions (Adams and Turner, 2010). Together, these results demonstrate that ethylene receptors in Arabidopsis have distinct functions, dependent on tissue and light context.

### The EIN3 and EBFs Mediate Light-Dependent Transcriptional Responses to Ethylene

The EIN3 TF is an essential mediator of ethylene response in hypocotyls of dark-grown seedlings, but its role is more complex in light-grown seedlings. The *ein3-1* mutant has ethylene-insensitive hypocotyl elongation in either light- or dark-grown hypocotyls (Chao et al., 1997; Smalle et al., 1997), suggesting that elongation responses to ethylene in the hypocotyl require EIN3 in a light-independent manner. EIN3 also regulates chlorophyll biosynthesis during the dark-to-light transition (Liu et al., 2017). However, the function of EIN3 in roots is light-dependent. In roots of dark-grown seedlings, double mutants between *ein3-1* and either *eil1-1* or *eil1-2* show no response to added ACC, while single mutants in *ein3* and *eil1* show partial response to this treatment (Alonso et al., 2003). In contrast, in roots of light-grown seedlings, *ein3-1*, *eil1-1*, and the double mutant all exhibit ACC-inhibition of root elongation and LR formation, and ACC stimulation of root hair formation (Harkey et al., 2018). A subset of ethylene-responsive transcripts from light-grown roots were identified as binding targets of EIN3 (Harkey et al., 2018) as reported by a DAP-Seq dataset (O’Malley et al., 2016), but many other transcripts were not direct EIN3 targets. These results are consistent with EIN3 and EIL1 controlling only a subset of ethylene responses in roots of light-grown seedlings. One example where there is light-dependent function of EIN3 is in regulation of *ACO2* transcript abundance. Upregulation of *ACO2* after ethylene treatment was lost in dark-grown *ein3-1* mutant seedlings (and EIN3 has been shown to bind to *ACO2 via* ChIP-Seq) (Chang et al., 2013). In contrast, in light-grown plants, that upregulation was present in the *ein3-1* single mutant, but was lost in both *ein3-1 eil1-1* and *ein3-1 eil1-2* double mutants (Lee et al., 2006), suggesting that EIL1 can compensate for EIN3 in regulating *ACO2* only in light-grown plants.

Recent results have suggested that differences in EIN3 function in the light and dark may be controlled at the level of turnover of this protein. Although *EIN3* transcript accumulation is not regulated by ethylene (Chao et al., 1997; Harkey et al., 2018), EIN3 and EIL1 protein accumulation is tightly controlled *via* ethylene-regulated turnover. In the absence of ethylene, EIN3 and EIL1 are ubiquitinated by EIN3-BINDING F-BOX PROTEIN1 and 2 (EBF1 and 2), two F-box proteins that act in SCF complexes, leading to EIN3 degradation. When ethylene levels rise, EBF1 and 2 are targeted for degradation in an EIN2-dependent manner, stabilizing EIN3 (Guo and Ecker, 2003; Gagne et al., 2004; Binder et al., 2007; An et al., 2010). EIN3 and EIL1 protein turnover is also regulated by crosstalk with light signaling *via* cryptochromes and HY5. The stimulation of hypocotyl elongation by ethylene in light-grown plants requires CRY1 or CRY2 (Vandenbussche et al., 2007), as well as HY5 (Yu et al., 2013). In darkness, CONSTITUTIVE PHOTOMORPHOGENESIS 1 (COP1), an integrator of light signaling, targets EBF1/2 and HY5 for ubiquitination and degradation, allowing EIN3 accumulation (Shi et al., 2016a), and preventing HY5-mediated inhibition of hypocotyl elongation. Movement of COP1 from the nucleus to the cytoplasm in light conditions allows HY5 to accumulate and inhibit growth. If ethylene signaling is activated in light conditions, EIN3 antagonizes HY5 and stimulates elongation by promoting nuclear localization of COP1, leading to HY5 degradation (Yu et al., 2013). The red light receptor PhyB also directly interacts with EIN3 and EBF1/2 after exposure to red light and enhances degradation of EIN3 (Shi et al., 2016b).

EIN3 regulation of *PIF3* and *ERF1*, which have antagonistic roles in regulating growth, constitutes one of the primary mechanisms driving the inverse hypocotyl responses to ethylene in light *versus* dark (Zhong et al., 2012). Both *PIF3* and *ERF1* are direct transcriptional targets of EIN3 (Chang et al., 2013). ERFs are stabilized by light, and they generally inhibit growth. EIN3 upregulates *ERF1* both in darkness and in light, but ERF1 effects on hypocotyl growth are only measurable under darkness, where other ERFs are absent. Conversely, *pif3* mutants are insensitive to ethylene-induced hypocotyl elongation in light, but not to hypocotyl inhibition in the dark (Zhong et al., 2012). PIFs generally promote elongation, and are destabilized in light, contributing to reduced elongation in light-grown seedlings. Transcriptional regulation of *PIF3* by ethylene *via* EIN3 is inconsequential in darkness, where many other PIFs are also active, but becomes significant under light, where other PIFs are degraded, and *PIF3* activation leads to increased hypocotyl growth. EBF1/2 also mediate red light-dependent degradation of PIF3 (Dong et al., 2017). EBFs can synergistically reduce PIF3 levels both directly, by promoting PIF3 degradation, and indirectly, by targeting EIN3 for degradation and thus reducing *PIF3* mRNA. This modulation of EIN3 and its targets by light enables complex responses to ethylene under different light contexts, such as opposite response in hypocotyl elongation. As discussed above light-dependent ethylene synthesis may also contribute to PIF3 stabilization and amplification of ethylene responses.

Downstream transcriptional effects of EIN3 and light signaling pathways cannot be completely disentangled. Recent work revealed that an *ein3 eil1* double mutant retains shade response, although ethylene-stimulated hypocotyl elongation is abolished (Das et al., 2016), suggesting that shade does not induce hypocotyl elongation by acting directly through the EIN3/EIL1 response pathway. The similar growth effects of ethylene and light are accompanied by many common transcriptional responses (Das et al., 2016). The COP1 effects on EIN3 targets are also complex. COP1 has been shown to increase EIN3 protein levels by targeting EBF1/2 for degradation in the dark (Shi et al., 2016a). In the light, ACC treatment and EIN3 overexpression lead to increased transcript levels of growth-promoting genes such as *YUCCA1* and *5*. This effect is lost in the dark but is restored in the *cop1-4* null mutant (Liang et al., 2012). This suggests that COP1 works by some mechanism downstream of EIN3 to fine tune expression of these particular genes so that they promote elongation in the light, but not in the dark. EIN3 and PIF1 transcriptionally regulate many of the same gene targets independently from one another, but mostly in the same direction (Jeong et al., 2016), and EIN3 and PIF1 pathways are each sufficient to maintain skotomorphogenesis (Shi et al., 2018). Overlapping transcriptional responses are also involved in EIN3/EIL1- and PIF3-mediated regulation of hypocotyl hook opening (Zhang et al., 2018). Downstream transcription factors, such as ERF72, may also have activity that is modulated by light to influence developmental responses (Liu et al., 2018). As described above, differential regulation of specific proteins, such as HY5, contributes to the opposing ethylene effects observed in light and dark (Smalle et al., 1997).

## Downstream Ethylene Transcriptional Effects Are Influenced by Light

A number of ethylene transcriptome studies have been performed with plants grown under a range of light conditions, revealing distinct transcriptional networks downstream of ethylene perception. We previously compared a dataset from dark-grown seedlings treated with ethylene (Chang et al., 2013) with another dataset from light-grown roots treated with ACC (Harkey et al., 2018). Both datasets used similar time points across a 24-h period after treatment, and we used the same statistical analysis of both datasets. However, we found limited overlap in differentially expressed (DE) genes (71 common genes out of 449 in the light-grown root dataset and out of 971 in the dark-grown seedling dataset). In principle, these changes could be explained by differences in light condition, tissue type, and/or method of elevating ethylene levels (ACC treatment vs. ethylene gas). This last possibility seems unlikely because all ACC responses were lost in the ethylene-insensitive *etr1-3* and *ein2-5* mutants (Harkey et al., 2018). Comparing a larger number of transcriptomic data sets is essential for more complete understanding of the light-dependent effects of ethylene on transcript accumulation.

To identify transcriptional responses to ethylene that are light- and tissue-specific, we looked for datasets that were suitable for a meta-analysis that could resolve differences and similarities in ethylene-responsive transcriptomes in the light and dark. We searched the Gene Expression Omnibus (GEO) for the term “ethylene.” Twenty-five datasets were identified in the original search based on treatment with ACC, ethylene, or with compounds that block ethylene synthesis (such as AVG), and/or mutations or transgenes that alter ethylene production or response. Many of these datasets were not usable because of dissimilar approaches or incomplete information. Five datasets were excluded due to insufficient information on experimental methods; another five used specific mutants or transgenic lines that were not found in any other dataset and did not include wild-type seedlings treated with ACC or ethylene. Although there were many datasets utilizing Col-0 and/or *ein2*, *ein3*, and *eil1* mutants in light and dark conditions, they used experimental methods, tissue types, or plants that were not developmentally matched. Seven additional datasets used 3- or 4-day-old whole dark-grown seedlings, while the remaining five datasets came from light-grown material using a variety of ages and tissue types. This highlights the need for future work that directly compares ethylene effects in light versus dark.

Ultimately, we identified three datasets with highly similar experimental methods and plant age in which transcript abundance was quantified after 4 h of ethylene or ACC treatment in roots (Stepanova et al., 2007; Feng et al., 2017; Harkey et al., 2018), and a fourth that provided an interesting comparison between ethylene treatment and shade treatment in hypocotyls or in cotyledons (Das et al., 2016). The most relevant differences between the three root datasets can be found in **Figure 3**, and further details on the process of identifying these datasets can be found in **Supplemental Datasheet 1**, along with a description of the experimental conditions used in each study. The fourth dataset was of particular interest because the authors compared the transcriptional effects of shade and ethylene in experimental conditions that were otherwise identical (Das et al., 2016). The authors noted that the effect of combined shade and ethylene on hypocotyl elongation was intermediate between the two individual treatments, consistent with ethylene and light signaling pathways sharing downstream signaling and/or effector components. However, samples treated with both ethylene and shade were not included in the transcriptomic analysis. Among genes that responded to ethylene and shade consistently and with the same direction of change, the authors found enrichments for annotations including hormone signaling, cell wall, and photomorphogenesis, among others, as well as two TFs, *AtHB28* and *IBL1*. Analysis of mutant and overexpression lines showed that *AtHB28* and *IBL1* are important for both shade and ethylene response (Das et al., 2016).

We developed a statistical pipeline to apply to all datasets used in our analysis to avoid discrepancies that might arise from differences in data analysis methods. We generated lists of DE genes that could more properly be compared to one another. (Note that this re-analysis results in DE lists that differ from those derived in the original publications.) For the three root datasets, we combined expression data from all three experiments into one master dataframe; both this dataframe and the Das et al. dataset were analyzed for differential expression using *limma* and other packages in R (Davis and Meltzer, 2007; R Core Team, 2014; Ritchie et al., 2015; Gu et al., 2016). Additional details of these analyses can be found in **Supplemental Datasheet 1**.

**Figure 2:**
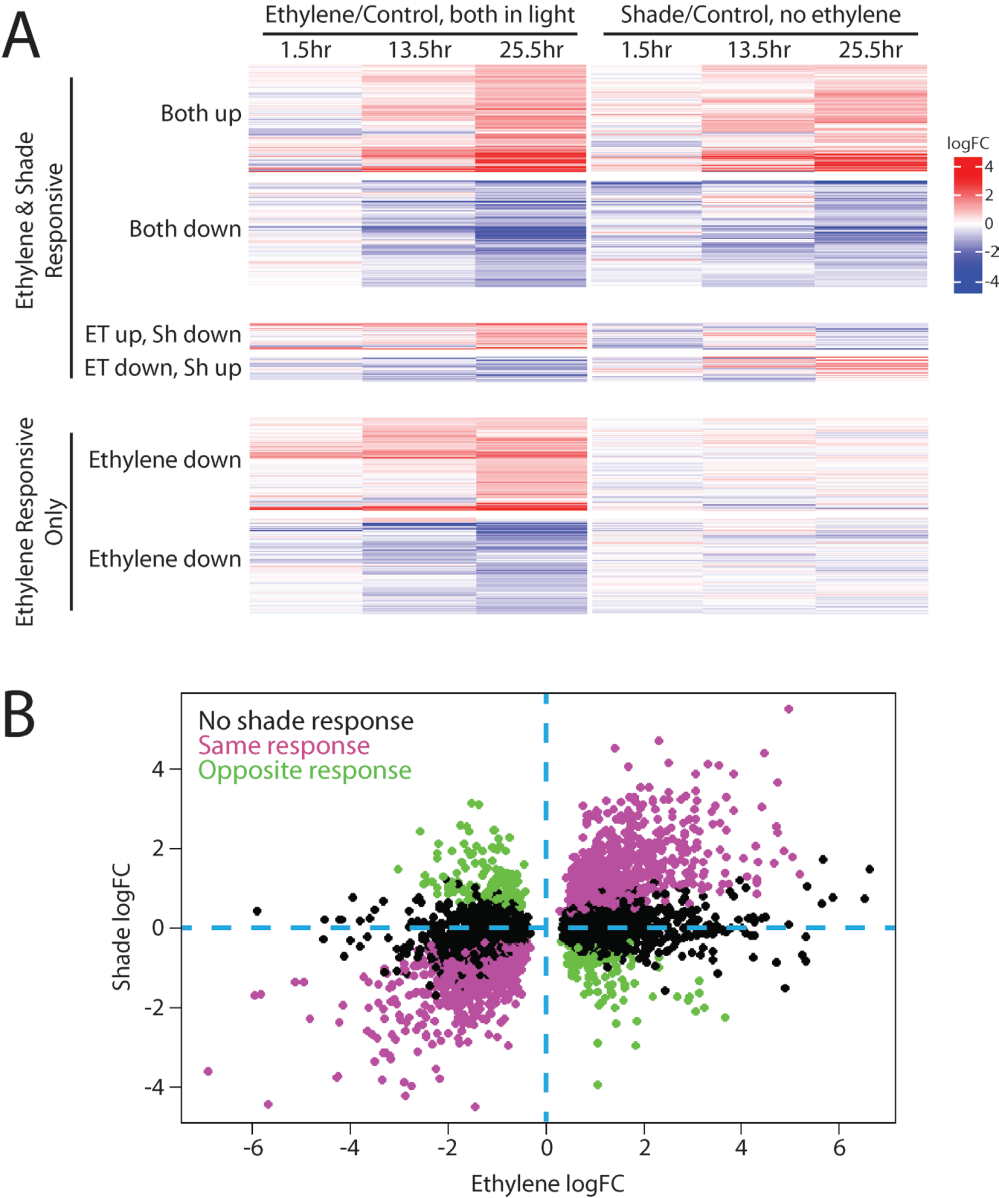
Ethylene and shade regulate many of the same genes in Arabidopsis hypocotyls. A transcriptional dataset in which seedlings were grown in the light and then either treated with ethylene or moved to shade (Das et al., 2016) and were refiltered as described in **Supplemental Datasheet 1**, revealing that many transcripts share both ethylene and shade regulation. **(A)** A heat map, generated using the Complex Heatmaps package in R (Gu et al., 2016), shows transcripts that had statistically significant responses to ethylene in at least one time point and how those transcripts responded to shade treatment. Most genes regulated by ethylene were also regulated by shade, with the majority changing in the same direction and a smaller subset changing in opposite directions, and with a limited number of transcripts showing no response to shade. **(B)** To better define the relationship between magnitude change in response to ethylene and light, the transcripts that showed significant changes in abundance with ethylene treatment in the 25.5 h sample (which showed most dramatic ethylene-induced abundance changes) were plotted as a function of their change in response to shading. Genes that were also regulated by shade in this dataset showed strong statistical correlations between ethylene logFC and shade logFC (positive for genes with the same direction of regulation (Pearson’s correlation, r = 0.89, p < 0.001), and negative for genes with the opposite direction of regulation (Pearson’s correlation, r = −0.87, p < 0.001).

To identify the entire overlap between ethylene and shade transcriptional responses in the Das et al. (2016) dataset, we used this data analysis pipeline. First, we identified the complete set of ethylene-responsive genes, and then queried their expression responses in the shade dataset. Compared to cotyledons, hypocotyls showed a greater response to ethylene, which is expected given the changes in hypocotyl growth that occur in etiolated seedlings treated with ethylene, described above, so we focused on that tissue type. Not surprisingly, of the 7,248 hypocotyl transcripts that showed a significant response to ethylene, more than half of those genes also showed a shade response (4,239; **Figure 2A**). The majority of these gene expression changes occurred in the same direction and with similar kinetics. Full results for all ethylene-responsive transcripts can be found in **Supplemental Datasheet 2**.

To better illustrate the relationship between ethylene and shade response, we plotted the log_2_ fold-changes in transcripts in response to ethylene against the fold-change in response to transition to shade (using the 25.5-h time point, which showed the most striking changes from the control) using the previously published transcript abundance values from Das et al. (2016). This graph highlights the strong correlation between ethylene response and shade response (**Figure 2B**). The correlation between the magnitude of change in response to ethylene and shade is statistically significant both for genes with the same direction of response (Pearson’s correlation, r = 0.89, p < 0.001) and in genes with the opposite direction of response (Pearson’s correlation, r = −0.87, p < 0.001). Dark- or shade-grown plants exhibit a different transcriptional landscape than their light-grown counterparts. Our analysis illustrates that many transcripts show similar responses to ethylene and shade; thus, studies that use dark-grown tissues to examine ethylene response will likely miss changes that occur only in light-grown plants.

**Figure 3:**
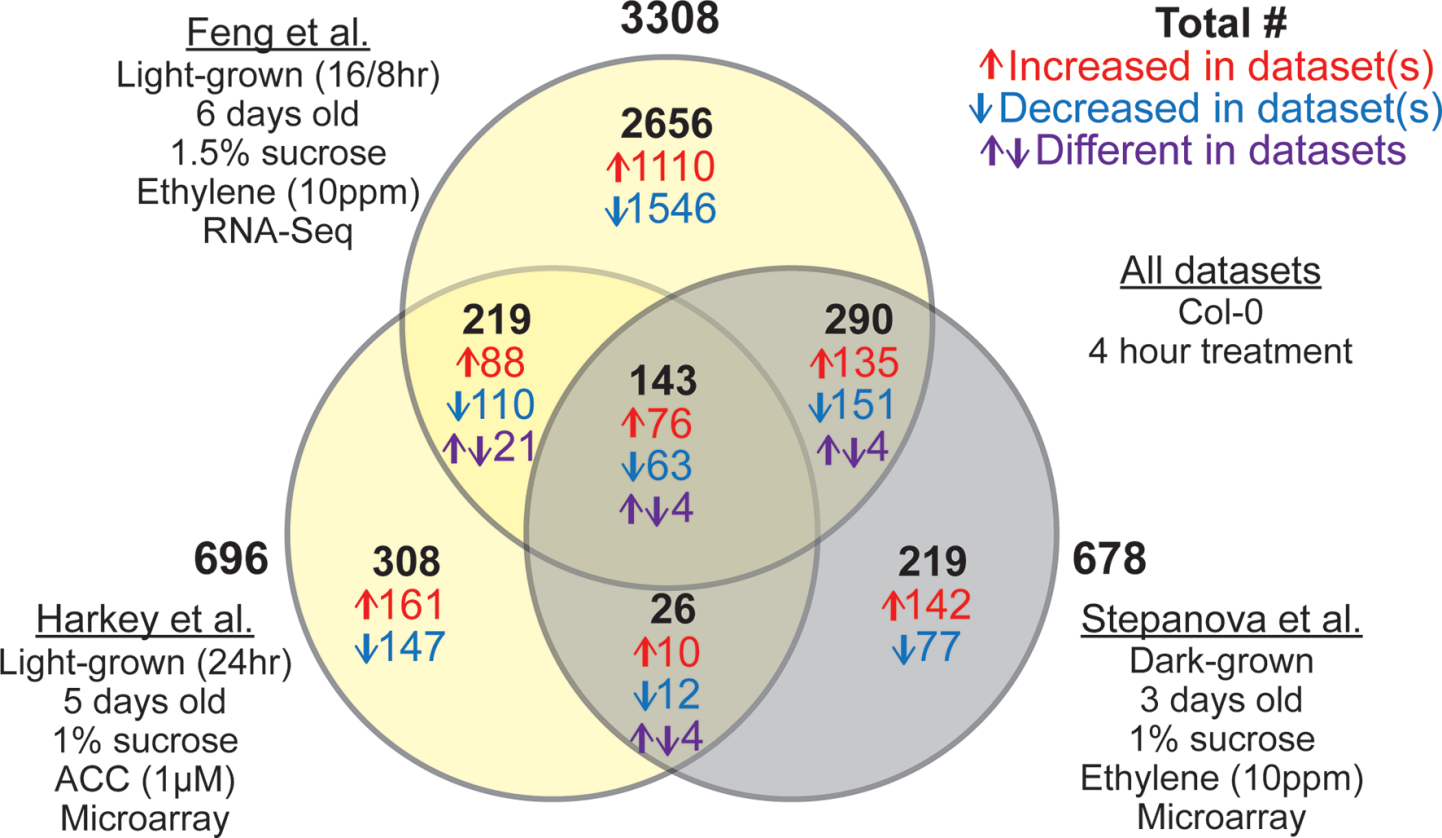
Three root-specific ethylene response datasets show light-dependent and light-independent overlaps. Venn diagram represents number of overlapping and non-overlapping DE genes between three root-specific transcriptomic datasets: Stepanova et al. (2007), Harkey et al. (2018), and Feng et al. (2017). Differences in experimental conditions are summarized under each dataset name. Details of the analysis can be found in **Supplemental Datasheet 1**. Once DE lists were generated for individual datasets, we compared the lists to find overlapping and non-overlapping genes. In the Venn diagram, the two light-grown datasets are represented in yellow, and the dark-grown dataset is represented in gray. The number of transcripts within each overlap are color coded, with the total in black, the number increasing in both or all three in red, the number decreasing in blue, and purple indicating transcripts that changed in different directions between datasets.

We performed a meta-analysis using the three root-specific ethylene-response datasets identified as sufficiently matched for comparison (Stepanova et al., 2007; Feng et al., 2017; Harkey et al., 2018) to screen for light-dependent and light-independent changes in ethylene-regulated transcript abundance. We used our new pipeline to reanalyze the root-specific transcriptomes to identify differences that are linked to the light environment of seedling growth. This analysis yielded interesting patterns of light-dependent and light-independent changes in transcript abundance that are summarized in a Venn Diagram in **Figure 3**. A list of all transcripts that showed significant responses to ethylene or ACC in at least one dataset and their magnitude of change can be found in **Supplemental Datasheet 2**. As expected, many more DE genes were identified in the RNA-seq dataset (Feng et al., 2017) than in the microarray-based datasets (Stepanova et al., 2007; Harkey et al., 2018), because RNA-Seq has a greater dynamic range. Although only 3% of the DE genes identified responded to ethylene in all three datasets, nearly a third (32%) were DE in two datasets. A number of genes were DE in the two datasets from light-grown seedlings (Feng et al., 2017; Harkey et al., 2018), but not in the dark (Stepanova et al., 2007), suggesting light-dependent regulation by ethylene. There was also substantial overlap (433 transcripts) between the two datasets that used ethylene treatment but differed in the presence of light during growth. We identified 169 transcripts in the overlap between the dark-grown ethylene dataset (Stepanova et al., 2007) and light-grown ACC dataset (Harkey et al., 2018). This number is greater than in our previously reported comparison of these two datasets (80, transcripts; Harkey et al., 2018), due to the common filtering used for both datasets in this meta-analysis. A surprising number of genes, however, were specifically regulated in one dataset, and not in the other two, despite the similarity of experimental techniques. These differences may be related to other conditions such as plant age (3, 5, or 6 days), light cycle (continuous light vs. 16 h light 8 h dark), or differences in media (e.g., sucrose concentration, which is also known to influence ethylene response; Gibson et al., 2001; Haydon et al., 2017; Yanagisawa et al., 2003). These results demonstrate the need for direct comparisons of ethylene effects under experimental conditions that vary only by light level.

In addition to the light-specific transcripts described above, this analysis identified a core set of 143 transcripts that responded to ethylene or ACC in all three datasets, regardless of light. Of these transcripts, 139 (97%) changed in the same direction in all treatments (**Figure 3**). This set of 139 genes with consistent direction of change should be considered the “gold standard,” for root ethylene response, much like a previously identified set of cytokinin-responsive genes from another meta-analysis (Bhargava et al., 2013). The full list of ethylene- or ACC-responsive genes from any dataset can be found in **Supplemental Datasheet 2** , with “gold standard” genes indicated.

A subset of the “gold standard” genes is summarized in **Table 1**. This group of 44 genes was chosen based on three criteria: the largest logFC values (in the positive or negative direction), known roles in ethylene synthesis or signaling (highlighted in red in **Table 1**), and/or known EIN3 targets based on DAP-Seq (O’Malley et al., 2016) and/or CHiP-Seq (Chang et al., 2013) analysis. Interestingly, most of the upregulated “gold” genes were identified as EIN3 targets by at least one method (72.4%), but very few downregulated “gold” genes were bound by EIN3 (6.3%). “Gold standard” genes also included a number of auxin-related genes (e.g., *SAUR76*, *SAUR8*, *IAA2*, and *IAA4/AUX2-11*), and genes involved in cell wall regulation (e.g., a pectin methylesterase inhibitor). Not surprisingly, the 139 transcripts were also enriched in gene annotations for cellular response to ethylene stimulus and negative regulation of the ethylene pathway.

**Table 1:** Selected gold standard transcripts regulated in all three datasets. The transcripts in red are all implicated in ethylene signaling or synthesis.

		logFC				EIN3 target?	
Gene ID	Gene Description	Feng 2017	Harkey 2018	Stepan. 2007	Ave	DAP-Seq	ChIP-Seq
AT5G19890	Peroxidase superfamily protein	6.20	3.55	6.49	5.41	YES	−
AT3G59900	ARGOS (Auxin-Regulated Gene Involved in Organ Size)	4.89	2.94	6.46	4.76	YES	
AT2G41230	ARGOS-LIKE2 (ARL2); (OSR1)	4.25	2.94	5.10	4.09	−	−
AT5G40590	Cysteine/Histidine-rich C1 domain family protein	3.86	4.05	4.36	4.09	−	YES
AT2G39980	HXXXD-type acyl-transferase family protein	4.63	2.96	4.25	3.95	−	YES
AT2G44080	ARGOS-LIKE (ARL)	4.37	2.43	3.90	3.57	YES	YES
AT5G53980	HOMEOBOX PROTEIN 52 (HB52)	4.15	1.97	3.34	3.15	−	YES
AT4G38410	Dehydrin family protein	2.99	2.77	3.68	3.14	−	−
AT5G02760	ARABIDOPSIS PP2C CLADE D 7 (APD7); (SSPP)	5.03	2.02	2.19	3.08	YES	YES
AT5G20820	SMALL AUXIN UPREGULATED RNA 76 (SAUR76)	3.61	2.61	2.75	2.99	−	YES
AT2G19590	ACC OXIDASE 1 (ACO1)	2.67	2.23	3.28	2.73	−	−
AT2G26070	REVERSION-TO-ETHYLENE SENSITIVITY1 (RTE1)	3.00	1.09	2.92	2.33	−	YES
AT3G23150	ETHYLENE RESPONSE 2 (ETR2)	2.21	1.80	2.79	2.27	YES	YES
AT3G25730	ETHYLENE RESPONSE DNA BINDING FACTOR3 (EDF3)	2.22	1.54	2.15	1.97	−	YES
AT1G04310	ETHYLENE RESPONSE SENSOR 2 (ERS2)	2.07	0.52	3.18	1.92	YES	YES
AT1G72360	ETHYLENE RESPONSE FACTOR 73 (ERF73); (HRE1)	1.66	2.14	1.68	1.83	−	−
AT5G25190	ETHYLENE AND SALT INDUCIBLE 3 (ESE3)	2.90	0.59	1.20	1.56	YES	YES
AT5G25350	EIN3-BINDING F BOX PROTEIN 2 (EBF2)	1.75	1.23	1.57	1.52	YES	YES
AT1G62380	ACC OXIDASE 2 (ACO2)	2.03	1.02	1.18	1.41	−	YES
AT2G40940	ETHYLENE RESPONSE SENSOR 1 (ERS1)	1.14	0.86	1.19	1.06	YES	YES
AT5G13330	RELATED TO AP2 6L (Rap2.6L)	1.36	0.84	0.79	1.00	YES	YES
AT5G03730	CONSTITUTIVE TRIPLE RESPONSE 1 (CTR1)	1.05	0.58	1.31	0.98	−	YES
AT5G04120	Cofactor-dependent phosphoglycerate mutase-like (dPGM) -	−5.49	−3.50	−3.52	−4.17	−	−
AT3G59370	Vacuolar calcium-binding protein-like protein	−4.33	−1.93	−2.91	−3.06	−	−
AT4G25250	PECTINMETHYLESTERASE INHIBITOR 4 (PMEI4)	−4.81	−1.81	−2.38	−3.00	−	−
AT2G20750	EXPANSIN B1 (EXPB1)	−3.57	−1.67	−3.29	−2.84	−	−
AT3G19320	Leucine-rich repeat (LRR) family protein	−4.19	−0.81	−3.42	−2.80	−	−
AT4G22460	Bifunctional inhibitor/lipid-transfer protein	−4.86	−1.52	−1.82	−2.73	−	−
AT2G18800	XYLOGLUCAN ENDOTRANSGLUCOSYLASE/HYDROLASE 21 (XTH21)	−4.46	−1.43	−1.66	−2.52	−	−
AT5G42590	CYTOCHROME P450, (CYP71A16); (MRO)	−2.00	−2.16	−2.30	−2.15	−	−
AT5G42580	CYTOCHROME P450, (CYP705A12)	−2.14	−2.12	−1.91	−2.06	−	−
AT5G24100	Leucine-rich repeat protein kinase family protein	−3.48	−1.02	−1.68	−2.06	−	−
AT3G25655	INFLORESCENCE DEFICIENT IN ABSCISSION (IDA)-LIKE 1 (IDL1)	−2.29	−2.15	−1.26	−1.90	−	−
AT4G02290	GLYCOSYL HYDROLASE 9B13 (GH9B13)	−2.64	−0.91	−2.15	−1.90	−	−
AT2G18980	Peroxidase superfamily protein	−3.70	−0.97	−0.74	−1.80	−	−
AT5G64620	VACUOLAR INHIBITOR OF FRUCTOSIDASE 2 (C/VIF2)	−2.98	−0.70	−1.57	−1.75	−	−
AT4G15290	CELLULOSE SYNTHASE LIKE 5 (CSLB5)	−3.11	−1.09	−1.04	−1.75	−	−
AT5G02230	Haloacid dehalogenase-like hydrolase (HAD) superfamily	−1.61	−1.23	−1.88	−1.57	YES	YES
AT5G59220	SENESCENCE ASSOCIATED GENE(SAG113); (HAI1)	−1.80	−1.22	−1.31	−1.44	−	YES
AT4G12730	FASCICLIN-LIKE ARABINOGALACTAN 2 (FLA2)	−2.01	−0.54	−0.93	−1.16	−	YES
AT1G08500	EARLY NODULIN-LIKE PROTEIN 18 (ENODL18)	−1.18	−1.32	−0.62	−1.04	YES	YES
AT4G30400	RING/U-box superfamily protein	−0.66	−0.57	−0.64	−0.62	−	YES

Within this group of 139 transcripts, we identified 13 core genes in ethylene signaling or synthesis whose levels increased in all three datasets (and in Das et al., 2016). This core gene set includes genes encoding TFs that participate in ethylene signaling (for example, EDF1, EDF3, EDF4, and several ERFs), negative regulators of the signaling pathway CTR1, EBF2 and ARGOS, and the ethylene receptors ETR2, ERS1, and ERS2. Thus, a core output of the ethylene response is upregulation of its own signaling pathway components including both positive and negative regulators of ethylene responses. The core set also includes transcripts encoding ethylene biosynthetic proteins. There is consistent upregulation of transcripts encoding the ACO enzymes, with *ACO1* and *ACO2* upregulated in all three datasets and *ACO3*, *ACO4*, and *ACO5* upregulated in two of the three datasets. *ACO2* was also upregulated by ethylene, although down-regulated in shade in Das et al. (2016). Interestingly, *ACS* transcript levels show less consistent positive regulation, showing no changes for any *ACS* gene in two datasets (Stepanova et al., 2007; Harkey et al., 2018) and changes in only two to four *ACS* transcripts (out of 11 family members) in two other data sets (Das et al., 2016; Feng et al., 2017). These results indicate that a positive feedback loop drives ethylene synthesis *via* upregulation of *ACO* expression, while *ACS* mRNA levels appear to be subject to a more complex control network, as discussed above (see *Light-Mediated Transcriptional Regulation of ACS and ACO*).

Finally, included in this comparison is an annotation of genes that are regulated by ethylene in dark-grown whole seedlings as detected by RNA-Seq (Chang et al., 2013) (as found in a separate column in the **Supplemental Datasheet 2**). Of the 77 up-regulated genes in the gold-standard list, 40 were also found to be sites of EIN3 binding while only 2 of the 62 down-regulated genes showed ethylene-regulated expression. Therefore, one can further refine these genes into root-specific and tissue-independent transcripts, using the detailed annotations in **Supplemental Datasheet 2**. Together, this meta-analysis reveals many candidate genes for conserved ethylene responses that are also induced by the ethylene precursor, ACC, and transcripts whose responses depend on light or tissue type. This information can allow formulation of a wealth of hypotheses that can be tested to further refine our understanding of ethylene signaling across plant development.

## Conclusions

As seedlings germinate, elongate through soil, and then emerge into light, they undergo profound changes in development. The importance of ethylene levels in controlling development is best understood in the early dark phases, but new studies that examine the role of ethylene during developmental transitions from dark to light or in light-dependent development are providing new insight into the functions of ethylene during seedling development. Recent studies have revealed novel mechanisms that modulate ethylene biosynthesis, including important transcriptional and post-translational regulatory strategies that control production of this hormone. The pathways that control ethylene response include central signaling proteins that function in ethylene response under all conditions, but also receptors and transcription factors with light- and developmental stage-specific functions. Comparison of genome-wide transcriptional datasets allows identification of candidate genes that contribute to all ethylene responses and other genes that may contribute to developmental outputs that are specific to the light environment. Together, light regulation of ethylene biosynthesis, signaling, and developmental response have far-reaching effects on a plant’s ability to adapt to the environment in early stages of development and throughout the life cycle. Understanding the mechanisms by which light and ethylene interact at the molecular and organismal levels is an important goal of future research.

## Author Contributions

AH performed the meta-analysis, drafted text, prepared figures, and edited the manuscript; GY drafted text, prepared figures, and edited the manuscript; DS prepared figures; AD and GM drafted text and edited the manuscript.

## Funding

This work was supported by grants from the US National Science Foundation to GM (MCB-1716279) and GY (MCB-1817286).

## Conflict of Interest Statement

The authors declare that the research was conducted in the absence of any commercial or financial relationships that could be construed as a potential conflict of interest.

## Supplementary Material

Supplemental Datasheet 1 Extended methods for meta-analysis of ethylene-regulated transcripts and ethylene- and shade-regulated transcripts

Supplemental Datasheet 2 Summary of differentially expressed transcripts from roots in response to ethylene or ACC and overlap of shade-and ethylene-regulated transcriptomes
