# Supplementary material for: Light Modulates Ethylene Synthesis, Signaling, and Downstream Transcriptional Networks to Control Plant Development
Source: Front Plant Sci. 2019 Sep 12;10:1094. doi: 10.3389/fpls.2019.01094 (PMC6751313; doi:10.3389/fpls.2019.01094)
Supplement: Supplemental Datasheet 2 — Summary of differentially expressed transcripts from roots in response to ethylene or ACC and overlap of shade-and ethylene-regulated transcriptomes [file DataSheet_2.pdf]

Supplemental Methods for Analysis of Transcriptomic Datasets

General Info **1**
Code repository 1
Analysis with limma 1
Das et al. 2016 Analysis **2**
Background 2
Experimental methods 2
Summary of analysis 3
Data Import 3
limma analysis 3
Root Ethylene and ACC Response Meta-analysis **4**
Background 4
Choice of datasets 4
Summary of Analysis 5
Prepare datasets for overlap 6
limma analysis 6

General Info

Code repository

For those interested, the full R markdown used to perform this analysis and generate figures is available on GitHub and on the Muday lab website.

Analysis with limma

limma stands for Linear Modeling for Microarray Data, and it is a package for the R programming environment. limma uses a linear modeling statistical approach for looking for significant differences between groups of samples, particularly suited for gene expression data such as microarray or RNA-Seq.

For details on using limma, see the limma user guides on Bioconductor here.

Das et al. 2016 Analysis

Background

The goal of this review and these analyses was to examine the way that light conditions interact with ethylene signaling. The Das et al. (2016) dataset was identified during our initial search for ethylene-related transcriptional datasets (described in more detail in the Root Meta-analysis section below), and was of interest due to its use of both ethylene and shade treatment under otherwise shared growth conditions.

The original study by Das et al. (2016) was intended to compare shade and flooding transcriptional responses. They used saturating ethylene treatment to mimic flood response, since localized ethylene levels increase when plants are submerged in water.

In this analysis, we examined the similarity between ethylene and shade response by asking what happens to ethylene-responsive genes in the shade-treated samples.

Experimental methods

Relevant experimental conditions used for generating data:

- All treatments used Col-0
- Plants were grown under a 16 hour light / 8 hour dark cycle
- At time of treatment, plants were approximately 2 days old
- Plants were either maintained in normal growth conditions (control), moved to shade, or treated with ethylene (1ppm)
- Samples were collected after 1.5, 13.5, and 25.5 hours of treatment
    - The 13.5 hour treatment was collected during the dark cycle
- Cotyledons and hypocotyls were separated and RNA was isolated for each sample
- Transcript abundance levels were measured using microarray

For additional information about experimental methods used for generating data, see Das et al. (2016)

Summary of analysis

The data used for this analysis is publically available at the Gene Expression Omnibus (GEO), accession number GSM2196539.

Data Import

The dataset was imported into R using the GEOquery package, and used to build a custom expression set (eSet) for use with limma. This data format connects the expression data (in this case microarray data) with the feature data (associated locus IDs) for each probe ID (in the case of microarray).

Part of this process included extracting the associated gene (or locus) IDs from the data file for the microarray platform used by Das et al. (2016), as seen here.

limma analysis

As the first step in this analysis, an Interquartile Range (IQR) filter was applied. The IQR is a measure of variability in a set of data, and is equal to the difference between the 25th and 75th percentiles. Any probe ID with an IQR less than 0.1 was removed for the remainder of the analysis.

For the linear modeling analysis, 2 comparisons were set up:

- Shade vs. control
- Ethylene vs. control

Each comparison was performed separately for each time point (1.5, 13.5, and 25.5 hours), and for each tissue type (hypocotyl and cotyledon), for a total of 12 comparisons.

Output for each comparison is given as a $\log_2$ fold change of the treatment over the matched control (logFC), with an associated p-value.

After these comparisons were performed, a Benjamini-Hochberg correction for multiple comparisons was performed. From there, differentially expressed (DE) genes were defined as having p-values < 0.05 and a logFC greater than 0.5 or less than -0.5.

Initially, any probe ID with a significant difference in any of the ethylene comparisons was kept, and compared to its response in shade samples. However, this analysis revealed that hypocotyl samples had a more dramatic response to ethylene than cotyledons, and most probe IDs which responded in cotyledon also responded in hypocotyl. Therefore, the rest of the analysis was performed for hypocotyl samples only. DE genes reported in Figure 2A have a significant ethylene response in at least one time point in hypocotyl. Genes reported Figure 2B have a significant ethylene response in the 25.5 hour time point.

Root Ethylene and ACC Response Meta-analysis

Background

The broad goal of the meta-analysis was to directly compare the transcriptional profile in samples treated with ethylene or its precursor, ACC, from light-grown and dark-grown plants that were otherwise as similar as possible, to identify transcriptional responses to ethylene that are light context-dependent and those that are similar across light growth conditions.

Choice of datasets

To identify datasets which might be useful for this meta-analysis, we searched the Gene Expression Omnibus (GEO) for the term "ethylene." After manually filtering the search results, we identified 25 datasets with treatment with ethylene or ACC, and/or with other relevant manipulations, such as mutants or transgenics with altered ethylene synthesis or signaling, or treatment with compounds that block ethylene synthesis (such as AVG). One of these datasets was the Das et al. (2016) dataset used above. 21 datasets were excluded for a variety of reasons:

- The methods were unclear (making it difficult to verify similarity to other datasets) or difficult to work with (e.g. Taconnat 2007 used an uncommon microarray platform)
- The lines used did not include a wild-type, and other lines were unsuitable for comparison
- There was no ethylene or ACC treatment, and where other treatments (such as inhibitors of ethylene synthesis or signaling) were used, no other datasets used comparable treatments
- There was otherwise no dataset with comparable methods (age of plants, tissue type, time of treatment, etc.) in the opposite light context that was suitable for comparison

A summary of the datasets excluded, and the reason(s) for exclusion:

**Dataset ID**	**Link to data**	**Insufficient Information on Methods**	**No WT**	**No ET/ACC treatment**	**No comparable dataset in light/dark**
Narusaka 2006	GSE4203	X		X	
Zhu 2010	GSE21762	X		X	
Alonso 2003	GDS414	X			
Qiao 2009	GSE14247	X			
Taconnat 2007	GSE7935	X			
Hall 2012	E-MEXP-3574		X		
Buchanan-Wollaston 2006	GSE5727			X	X

Cheng 2009	GSE12715			X	X
De Grauwe 2007	GSE6150			X	X
Dubois 2013	GSE45830			X	X
Heyman 2013	GSE48836			X	
Lin 2010	GSE20897			X	
Tsai 2014	GSE51767			X	X
Tsuchisaka 2009	GSE14496			X	
Zhong 2009	GSE18631			X	
Chang 2013	SRA063695				X
Olmedo 2006	GSE5174				X
Zhang 2016 NatCom	GSE77395				X
Zhang 2016 PLOS	GSE83573				X
Zhang 2017	GSE83214				X
Zhang 2018	GSE101762				X

The datasets ultimately chosen for the analysis were from Feng et al. (2017), Harkey et al. (2018), and Stepanova et al. (2007). All three datasets had similar methods:

- Young plants (3-5 days)
- Root tissue only
- Treatment with ethylene or ACC for 4 hours

The datasets differ in the light conditions under which plants were grown:

- Feng et al. grew plants under a 16 hour light / 8 hour dark cycle
- Harkey et al. grew plants under continuous light
- Stepanova et al. grew plants under continuous dark

This made them the best candidates for direct analysis. However, Feng et al. used RNA-Seq, while Harkey et al. and Stepanova et al. both used microarray, so a large part of the meta-analysis involved reconciling the data between these two formats, as described below.

Additional information on methods used to generate each dataset can be found in their corresponding publications.

Summary of Analysis

The data used in these analyses can be found at the following locations:

- Feng et al., GEO GSE107699
- Harkey et al., GEO GSE84446

- Stepanova et al., GEO GSE7432
- Affymetrix ATH1 probe annotations from TAIR

Prepare datasets for overlap

One issue with comparing microarray and RNA-Seq datasets with one another is the way they handle gene IDs. For Affymetrix microarrays, data is given for microarray probes, which each have their own unique ID (the probe ID). Most probe IDs are associated with one locus, or gene ID (the AGI, formatted ATxGxxxxx), but because of the probe design, some are associated with two or more gene IDs. Additionally, one gene ID may be associated with multiple probe IDs.

For RNA-Seq, data is sometimes (as in the case of the Feng dataset) given for individual gene models (ATxGxxxxx.X), so there may be multiple gene models for each gene ID.

The goal of this analysis was to eventually compare genes across all datasets, so it was important to arrive at a dataset with exactly one row of data for each unique gene ID. The strategy used was to:

- For microarray probe IDs with multiple associated gene IDs, a new row with the same data was created for each additional gene ID associated with that probe ID
    - I.e. If a probe ID has 3 associated gene IDs, we would create 3 rows with identical data, but a different gene ID for each
- For RNA-Seq, the gene model number was dropped, leaving only the gene ID
- In both cases, this resulted in duplicate gene IDs with different data. For theses gene IDs, the row with the highest variation across the data, measured by the interquartile range (IQR), was kept, so that the final result is one set of data per each unique gene ID.

The Harkey and Stepanova datasets use the same Affymetrix platform, and so this approach left the same exact unique gene IDs for both of these lists. However, the Feng RNA-Seq data had more gene IDs represented. For the purposes of this analysis, genes with data in only one dataset don't provide any useful information, so only the overlap in the unique gene IDs from both RNA-Seq and microarray were kept in the final dataset.

The RNA-Seq data was log~2~ transformed for consistency with the microarray data, which is log~2~ transformed as part of the Affymetrix data processing. Finally, the data from all three datasets was combined into one data frame for the remainder of the analysis.

limma analysis

As the first step in this analysis, an Interquartile Range (IQR) filter was applied. The IQR is a measure of variability in a set of data, and is equal to the difference between the 25th and 75th percentiles. Any probe ID with an IQR less than 0.1 was removed for the remainder of the analysis.

For the linear modeling analysis, 3 comparisons were set up:

- Ethylene vs. control in Feng et al. and Stepanova et al.
- ACC vs. control in Harkey et al.

Output for each comparison is given as a $\log_2$ fold change of the treatment over the matched control (logFC), with an associated p-value.

After these comparisons were performed, a Benjamini-Hochberg correction for multiple comparisons was performed. From there, differentially expressed (DE) genes were defined as having p-values < 0.05 and a logFC greater than 0.5 or less than -0.5. Genes represented in Figure 3 were DE in at least one dataset, and genes represented in Table 1 were DE in all three datasets.
